# Real-time Tracking and Classification of Tumor and Nontumor Tissue in Upper Gastrointestinal Cancers Using Diffuse Reflectance Spectroscopy for Resection Margin Assessment

**DOI:** 10.1001/jamasurg.2022.3899

**Published:** 2022-09-07

**Authors:** Scarlet Nazarian, Ioannis Gkouzionis, Michal Kawka, Marta Jamroziak, Josephine Lloyd, Ara Darzi, Nisha Patel, Daniel S. Elson, Christopher J. Peters

**Affiliations:** 1 Department of Surgery and Cancer, Imperial College London, London, United Kingdom; 2 Hamlyn Centre, Imperial College London, London, United Kingdom; 3 Histopathology Department, Imperial College NHS Trust, London, United Kingdom

## Abstract

**Importance:**

Cancers of the upper gastrointestinal tract remain a major contributor to the global cancer burden. The accurate mapping of tumor margins is of particular importance for curative cancer resection and improvement in overall survival. Current mapping techniques preclude a full resection margin assessment in real time.

**Objective:**

To evaluate whether diffuse reflectance spectroscopy (DRS) on gastric and esophageal cancer specimens can differentiate tissue types and provide real-time feedback to the operator.

**Design, Setting, and Participants:**

This was a prospective ex vivo validation study. Patients undergoing esophageal or gastric cancer resection were prospectively recruited into the study between July 2020 and July 2021 at Hammersmith Hospital in London, United Kingdom. Tissue specimens were included for patients undergoing elective surgery for either esophageal carcinoma (adenocarcinoma or squamous cell carcinoma) or gastric adenocarcinoma.

**Exposures:**

A handheld DRS probe and tracking system was used on freshly resected ex vivo tissue to obtain spectral data. Binary classification, following histopathological validation, was performed using 4 supervised machine learning classifiers.

**Main Outcomes and Measures:**

Data were divided into training and testing sets using a stratified 5-fold cross-validation method. Machine learning classifiers were evaluated in terms of sensitivity, specificity, overall accuracy, and the area under the curve.

**Results:**

Of 34 included patients, 22 (65%) were male, and the median (range) age was 68 (35-89) years. A total of 14 097 mean spectra for normal and cancerous tissue were collected. For normal vs cancer tissue, the machine learning classifier achieved a mean (SD) overall diagnostic accuracy of 93.86% (0.66) for stomach tissue and 96.22% (0.50) for esophageal tissue and achieved a mean (SD) sensitivity and specificity of 91.31% (1.5) and 95.13% (0.8), respectively, for stomach tissue and of 94.60% (0.9) and 97.28% (0.6) for esophagus tissue. Real-time tissue tracking and classification was achieved and presented live on screen.

**Conclusions and Relevance:**

This study provides ex vivo validation of the DRS technology for real-time differentiation of gastric and esophageal cancer from healthy tissue using machine learning with high accuracy. As such, it is a step toward the development of a real-time in vivo tumor mapping tool for esophageal and gastric cancers that can aid decision-making of resection margins intraoperatively.

## Key Points

**Question:**

Can the development of a diffuse reflectance spectroscopy (DRS) probe aid cancer margin assessment in esophageal and gastric cancer surgery?

**Findings:**

In this validation study including 37 patients, the development of a DRS probe and tracking system showed that machine learning can provide real-time discrimination of normal and cancer tissue with high diagnostic accuracy of 93.9% and 96.2% for gastric and esophageal specimens, respectively.

**Meaning:**

This study highlights the potential for the DRS system to be used intraoperatively to aid cancer margin assessment in real time.

## Teaser

This validation study evaluates whether diffuse reflectance spectroscopy on gastric and esophageal cancer specimens can differentiate tissue types and provide real-time feedback to the operator.

## Introduction

Esophageal and gastric cancers are associated with poor prognosis, with 5-year overall survival of patients estimated to be between 15% and 25%.^1,2^ This is despite an improvement in survival over the last half century, owing to advances in modern neoadjuvant therapies and the implementation of new surgical approaches.^3,4,5,6,7,8,9,10^

The use of neoadjuvant treatments makes accurate preoperative tumor mapping challenging because of the formation of scar tissue over healthy areas adjacent to the tumor.^11,12^ Mapping tumor margins accurately is of particular importance for achieving a negative resection margin (R0), which is the goal of curative cancer resection. Residual disease has been shown to nearly double the risk of dying from esophageal cancer.^13,14,15,16^ Similarly, a positive resection margin in gastric cancer has been identified as an independent risk factor for reduced overall and recurrence-free survival.^17^

The lack of accurate tumor mapping tools and anatomical limitations of the upper abdomen and thorax lead to difficulties in achieving negative resection margins.^18,19^ This is compounded by the lack of consensus on optimal margin length^20,21^ and attempts to preserve as much healthy tissue as possible. Therefore, there is a pertinent need for developing accurate technologies and tools for preoperative or intraoperative tumor mapping.

A criterion standard for any intraoperative tissue assessment is frozen section analysis. This technique has been shown to have a diagnostic accuracy of 93%, a sensitivity of 67%, and a specificity of 100% in gastric and esophageal adenocarcinoma.^22^ However, frozen sections are limited by a slow processing time and a finite number of distinct areas that can be sampled, precluding a full resection margin assessment in real time.^23^

These challenges can potentially be addressed by using multispectral optical probes, which have been previously shown to have high sensitivity and specificity (greater than 90%) for discriminating between normal and cancer tissue.^24^ Diffuse reflectance spectroscopy (DRS), a point-based spectroscopy technique, is particularly promising because of its low cost and ease of use. Compared with sophisticated microendoscopic probes, DRS is easier to implement, as it does not require lasers or advanced magnification optics.^25^ It can be used to differentiate tissue classes through the quantification of otherwise invisible tissue and cellular changes at microscale and nanoscale levels, present at both early and late stages of malignant transformation.^25^ The DRS system has been applied to ex vivo differentiation of tumors from healthy surrounding tissues across multiple malignancy types, such as colorectal, lung, and breast cancers.^26,27,28,29^ Nevertheless, to our knowledge, although DRS has been used in the context of premalignant upper gastrointestinal disease,^30,31^ its use in esophageal and gastric cancer has not been fully explored. Therefore, the primary aim of this validation study was to use DRS to accurately differentiate cancer tissue from normal tissue in ex vivo gastric and esophageal specimens and to develop a real-time classification system that is able to provide live feedback about tissue type to the surgeon to aid margin assessment intraoperatively.

## Methods

### Study Setting

Consecutive patients undergoing esophageal or gastric cancer resection were prospectively recruited into the study between July 2020 and July 2021 at Hammersmith Hospital in London, United Kingdom. Ex vivo tissue specimens were included for patients undergoing elective surgery for either esophageal carcinoma (adenocarcinoma or squamous cell carcinoma) or gastric adenocarcinoma. The study was performed with approval from the Harrow Research Ethics Committee, and all participants provided written informed consent.

### DRS Instrumentation

**Figure 1.**
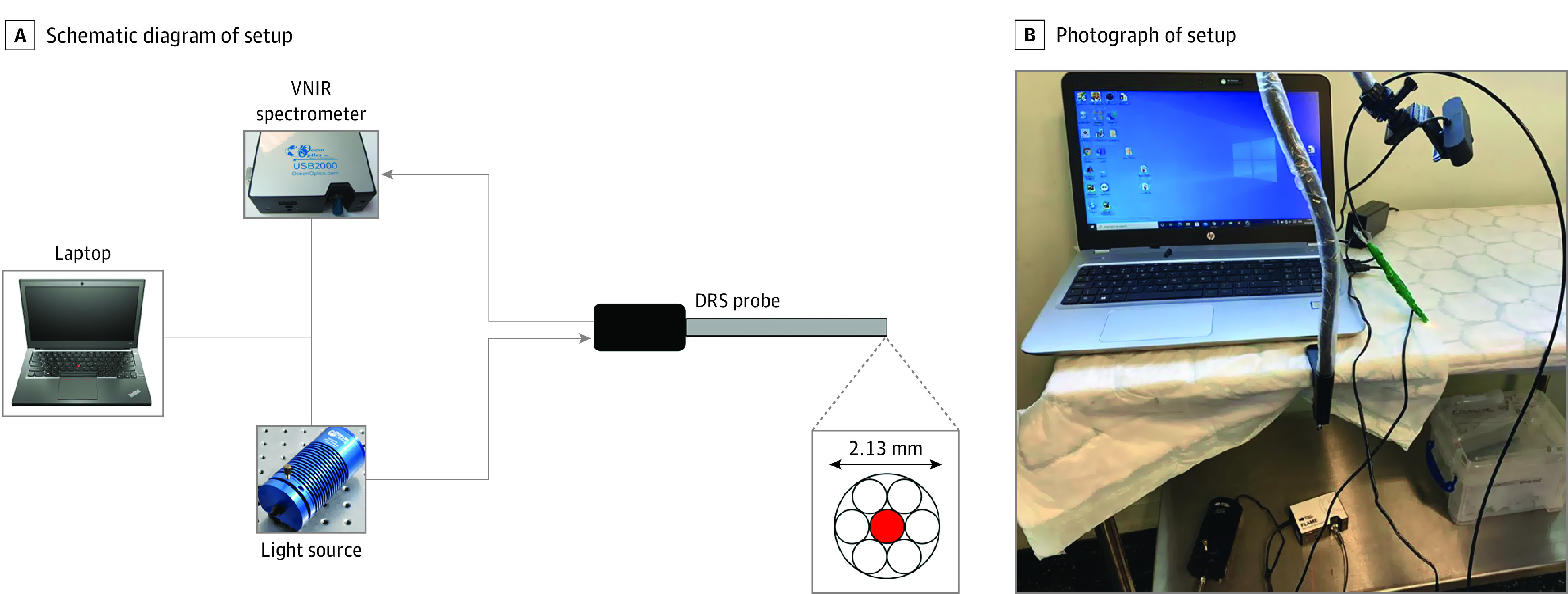
Diffuse Reflectance Spectroscopy (DRS) Instrumentation for Ex Vivo Data Acquisition The DRS probe was connected to both the light source (HL-2000-HP; Ocean Optics) and the spectrometer (USB4000; Ocean Optics) to allow for data acquisition and sample illumination. All electronic devices communicated with proprietary software designed with Python version 3.6 (Python Software Foundation) on the laptop.^32^ VNIR indicates visible and near-infrared.

A handheld reflection fiber probe (QR600-7-SR-125F; Ocean Optics) was used to collect the DRS spectra. The probe contained 6 peripheral illumination fibers together with a central light collection fiber, each measuring 600 micrometers in diameter, within a 0.125-inch ferrule in a cylindrical configuration. The instrumentation and setup have been previously described.^32^ A schematic representation and photograph of the instrumentation for data acquisition is shown in Figure 1.

### Real-time Tracking of DRS Probe

The main limitation of the DRS probe was that it did not leave any marks on the tissue being scanned. To overcome this limitation and localize the optical biopsy sites on the specimen, an optical tracking method was used, as described in previous work.^32^ Briefly, to track the DRS probe, a color marker was chosen based on the color distribution of biological tissue in the hue saturation value (HSV) color space. Green plastic tape was wrapped around the distal end of the DRS probe and used as a marker. Tracking of the probe was achieved using a Kalman filter.^33^ The exact probe coordinates at each sampling point were recorded. In this way, the localization of the probe tip was known in real time.

### Data Acquisition Protocol

Data acquisition was performed within 15 minutes of resection of the surgical specimen and lasted approximately 10 minutes. An overview image and a video recording were captured by an RGB camera, which allowed for tracking of the tip of the DRS probe and mapping of sampling locations in real time, as previously described.^32^

The DRS probe was then used to scan the outer wall of the tissue, and spectral data were captured. Suspected normal and cancerous tissues were identified by visual and haptic inspection by a surgeon. During the spectral data acquisition, the DRS probe was tracked in real time on the live video feedback from the camera. Normal tissue measurements were taken near the resection margins, as far away from the suspected cancerous location as possible. Sampling at the resection margin itself was avoided to prevent any interaction with clips, sutures, or staples. Staples and clips would be expected to affect the spectral data because of their reflective properties (eFigure 1 in the Supplement).

The DRS probe acquired 80 spectra per second. Twenty spectra per point of tissue sample were acquired and averaged, and the mean spectrum was displayed in real time. A minimum of 200 point-based spectral measurements per tissue type (normal or tumor) were collected, depending on the size of the sampled region.

### Histopathology Correlation

After the acquisition of all spectra, the suspected tumor location was painted with yellow tissue dye (Cancer Diagnostics Inc) to allow for correlation with histopathological analysis, which was used as a reference test. Samples were then placed into formalin and sent to the histopathology department, where they were processed according to standard protocols. Macroscopic photos of the whole specimen, as well as slices, were taken.

Once the painted area on the tissue samples was confirmed as the tumor, the corresponding images were correlated with the video frames and recordings containing the tracked positions of the suspected tumor (eFigure 2 in the Supplement). The video frames were then manually labeled as tumor or nontumor tissue. These labels were considered ground truth labels for the training of machine learning classifiers.

For specimens in which the suspected tumor area was confirmed by the pathologist as fully regressed tumor following neoadjuvant therapy (T0), only normal tissue data collected for the specimen were analyzed. Tracked locations on the specimen, which could not be confirmed by the histopathological analysis, were excluded.

### Classification

Data from all patients were combined into either esophagus or stomach data sets. Ex vivo labeling errors of spectral data were identified and excluded from further analysis by reviewing videos of real-time data acquisition retrospectively and analyzing the DRS probe position over the tissue areas. Probe contact artifacts (eg, lack of probe contact with the tissue) were automatically removed. To account for interpatient, background light, and signal quality variability, spectral normalization and noise reduction were performed, using white reflectance standard and dark field readings. An automated method was implemented for outlier (eg, erroneous measurements or air interference) detection and removal.

To reduce overfitting and improve classification accuracy, feature extraction was performed on the esophagus and stomach data sets. The 2 most prominent peaks of a spectrum were selected. The mean intensity values around the spectral range of these peaks and the mean intensity value across the overall spectral range were derived. Moreover, another 3 feature extraction techniques were evaluated, namely the permutation feature importance,^34^ the recursive feature elimination,^35^ and the Boruta method.^36^

Data were divided into training and testing sets using the repeated stratified *k*-fold cross-validation method, with 5 folds and 5 repeats. Binary classification into normal and tumor tissue was performed using various supervised machine learning classifiers, such as linear support vector machine, multilayer perceptron, light gradient boosting machine, and extreme gradient boosting (XGB).

### Statistical Analysis

Machine learning classifiers were evaluated in terms of sensitivity, specificity, overall accuracy, and the area under the curve (AUC). Overall accuracy was calculated as the proportion of correctly identified spectra over a total number of spectra. Receiver operating characteristic (ROC) curves were plotted. Python version 3.6 (Python Software Foundation) was used for machine learning classification, MATLAB version R2020b (MathWorks) was used for data processing, and R version 4.0 (The R Foundation) was used for visualization and statistical analysis.

## Results

### Cohort Characteristics

A total of 37 patients were recruited into this study. Three patients were excluded due to incomplete data acquisition, resulting in 34 patients being included in the final analysis. Of these, 22 (65%) were male, and the median (range) age was 68 (35-89) years. Additional patient characteristics can be seen in Table 1. A total of 19 patients (56%) underwent an esophagectomy, and 15 (44%) underwent a gastrectomy procedure. Most of the patients (27 [79.4%]) had cancer demonstrated to be adenocarcinoma at histology, with 2 (5.9%) being squamous cell carcinoma. At pathological staging, 5 of 34 tumors (14.7%) were found to be regressed or showed no tumor presence.

**Table 1.** Cohort Characteristics

Characteristic	No. (%)		
	All patients	Patients with cancer	
		Gastric	Esophageal
Age, median (range). y	68 (35-89)	61 (43-89)	68 (35-80)
Sex			
Female	12 (35.3)	4 (26.7)	8 (42.1)
Male	22 (64.7)	11 (73.3)	11 (57.9)
Procedure			
Esophagectomy	19 (55. 9)	0	19 (100)
Gastrectomy	15 (44.1)	15 (100)	0
Tumor histology			
Adenocarcinoma	27 (79.4)	12 (80.0)	15 (78.9)
Squamous cell carcinoma	2 (5.9)	2 (13.3)	0
Tumor stage (TNM staging)			
0	5 (14.7)	1 (6.7)	4 (21.1)
1	6 (17.7)	5 (33.3)	1 (5.2)
2	4 (11.8)	3 (20.0)	1 (5.2)
3	15 (44.1)	4 (26.7)	11 (57.9)
4	4 (11.8)	2 (13.3)	2 (10.6)
Neoadjuvant therapy			
Chemotherapy	24 (70.6)	8 (53.3)	16 (84.2)
Chemoradiotherapy	1 (2.9)	0	1 (5.3)
Radiotherapy	1 (2.9)	1 (6.7)	0
None	8 (23.5)	6 (40.0)	2 (10.5)

### Data Set Summary

**Figure 2.**
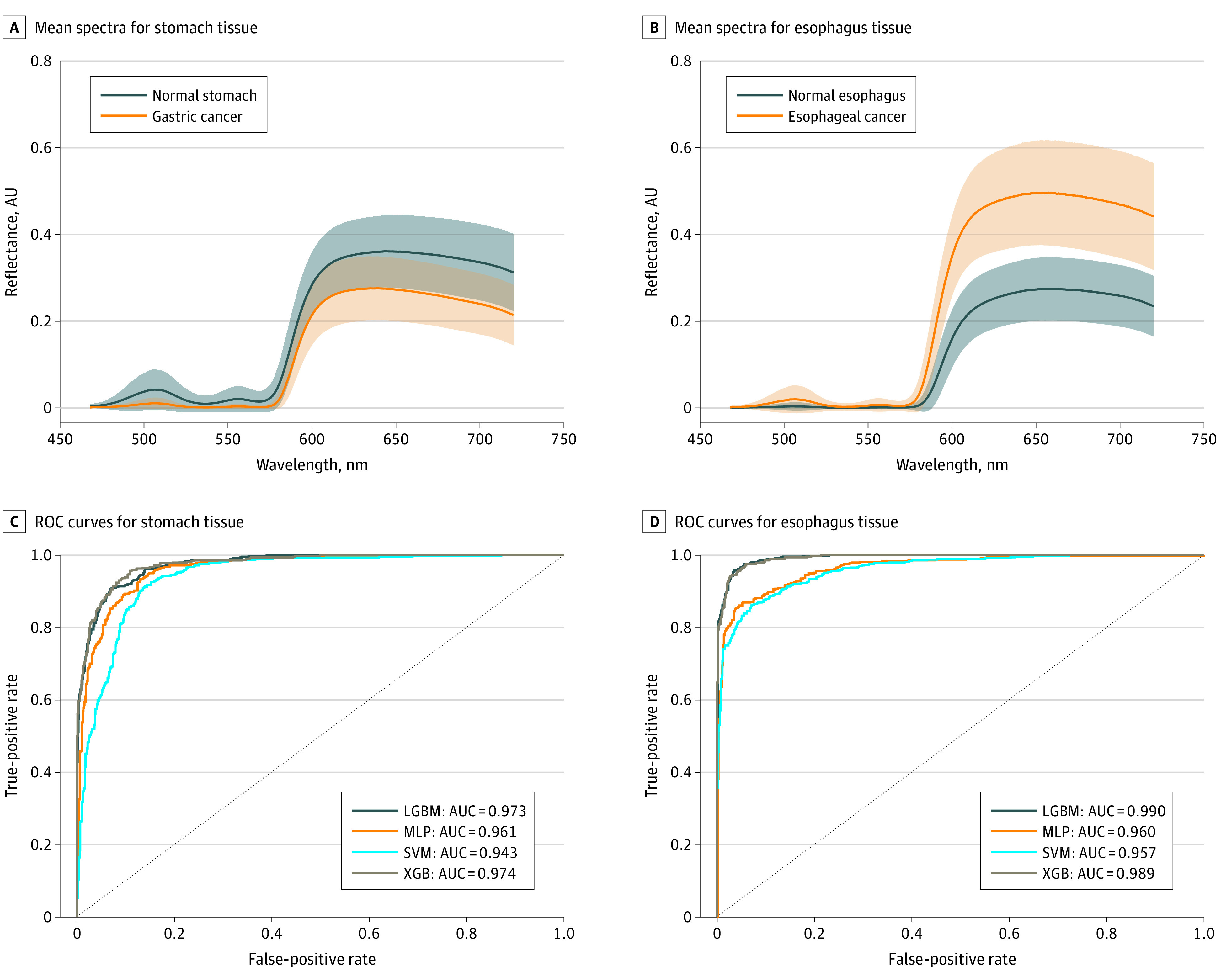
Mean Spectra of the Stomach and Esophagus A and B, Sample size of 34 patients, including 7937 mean spectra for stomach tissue and 6160 mean spectra for esophagus tissue. C and D, Receiver operator characteristic (ROC) curves for stomach and esophagus tissue. AUC indicates area under the receiver operating characteristic curve; LGBM, light gradient boosting machine; MLP, multilayer perceptron; SVM, support vector machine; XGB, extreme gradient boosting.

Overall, 23 distinct sets of normal stomach data, 16 sets of normal esophagus data, 10 sets of gastric cancer data, and 10 sets of esophageal cancer data were recorded. A total of 5496 mean spectra were collected for the normal stomach set, 2441 mean spectra for gastric cancer set, 3677 mean spectra for normal esophagus set, and 2483 mean spectra for esophageal cancer set. Each processed mean spectrum contained 505 equally spaced intensity measurements in the 468 to 720 nanometer spectral range (resolution, 0.5 nanometers), with data from the 420 to 468 nanometer and the 720 to 1000 nanometer spectral ranges excluded following interim analysis. The means of all spectra for each of the tissue classes are shown in Figure 2.

### Tissue Classification

Results of the classification for stomach and esophagus spectral data are presented in Table 2. The XGB classifier was the best-performing machine learning algorithm for both stomach and esophagus tissue. Compared with the other algorithms tested in this study, the XGB model generally showed better performance in terms of accuracy, sensitivity, and specificity for normal vs cancer tissue, achieving a mean (SD) overall diagnostic accuracy of 93.86 (0.66) for stomach tissue and 96.22 (0.50) for esophageal tissue. The mean (SD) sensitivity and specificity of the classifier were 91.31% (1.5) and 95.13% (0.8), respectively, for stomach tissue and 94.60% (0.9) and 97.28% (0.6) for esophageal tissue. The mean (SD) AUC of the XGB classifier was 0.974 (0.002) for stomach tissue and 0.989 (0.001) for esophagus tissue. The ROC curves are shown in Figure 2. In addition, the XGB model showed a much higher computation speed than all the other algorithms used in this study because of its inherent parallel processing, with only 3.5 seconds over both training and validation phases.

**Table 2.** Performance Metrics for the Spectral Data Classification Using Supervised Machine Learning and Permutation Feature Importance for the Feature Selection^a^

Classifier	Mean (SD)			
	Accuracy	Sensitivity	Specificity	AUC
**Stomach**				
XGB	0.938 (0.006)	0.913 (0.015)	0.951 (0.008)	0.974 (0.002)
LGBM	0.9363 (0.007)	0.910 (0.016)	0.949 (0.007)	0.973 (0.002)
MLP	0.900 (0.009)	0.862 (0.044)	0.917 (0.014)	0.961 (0.005)
SVM	0.876 (0.008)	0.834 (0.012)	0.895 (0.010)	0.943 (0.005)
**Esophagus**				
XGB	0.962 (0.005)	0.946 (0.009)	0.972 (0.006)	0.989 (0.001)
LGBM	0.962 (0.026)	0.943 (0.009)	0.974 (0.005)	0.990 (0.001)
MLP	0.919 (0.029)	0.851 (0.040)	0.959 (0.029)	0.960 (0.004)
SVM	0.883 (0.009)	0.821 (0.043)	0.923 (0.023)	0.957 (0.005)

Abbreviations: AUC, area under the receiver operating characteristic curve; LGBM, light gradient boosting machine; MLP, multilayer perceptron; SVM, support vector machine; XGB, extreme gradient boosting.

^a^ Data showing the ability of multiple classifiers to detect normal vs cancerous tissue presented as mean (SD). Overall accuracy is calculated as the proportion of correctly identified spectra over the total number of spectra.

**Figure 3.**
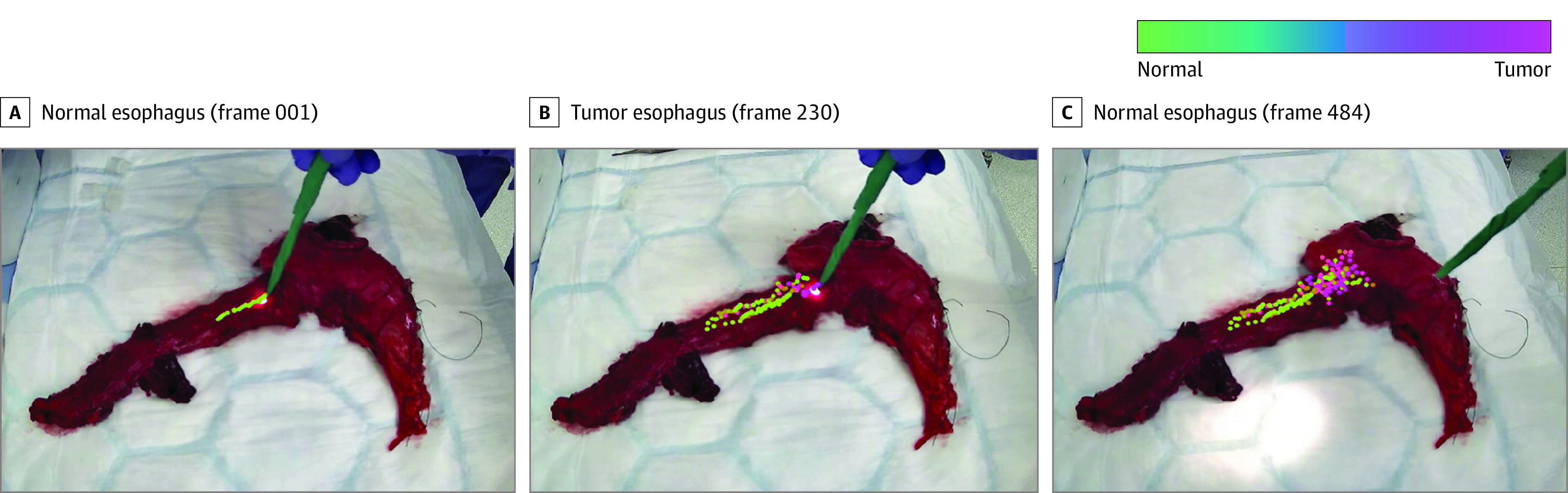
Three Illustrations of Real-time Diffuse Reflectance Spectroscopy Probe Tracking and Classification at Different Frames During Scanning of an Esophageal and Gastric Ex Vivo Tissue Specimen Real-time tracking at each optical biopsy site coupled with the binary classification of each site on an esophageal and gastric specimen. Tissue type highlighted on the screen in real time.

Real-time tissue classification was achieved and presented on the user interface when using the DRS probe. Real-time tracking at each optical biopsy site coupled with the binary classification of each site was visualized as either normal (100% green) or tumor tissue (100% pink) using a graduated color map. Tissue type was highlighted on the screen in real time during sampling, as highlighted in Figure 3.

## Discussion

In this ex vivo validation study, we show that, with the use of a DRS probe together with a tracking system, machine learning can provide real-time discrimination of normal stomach tissue from gastric cancer as well as normal esophagus tissue from esophageal cancer with an overall accuracy of 93.9% and 96.2%, respectively. The machine learning classifier performed with an AUC of 0.974 for stomach tissue and 0.989 for esophagus tissue. Moreover, we were able to develop a real-time classification system, providing live feedback on screen to highlight the tissue type being sampled.

Intraoperative margin mapping techniques have become increasingly common in the last 10 years. These include rapid evaporative ionization mass spectrometry,^37^ ultrahigh-resolution optical coherence tomography,^38^ bio-impendence measurement,^39^ and fluorescence-guided imaging.^40^ However, these techniques have either failed to or have not yet translated into routine clinical practice because, while potentially reducing the time required for definitive tissue classification, they either cannot provide real-time data, do not fit into the surgical workflow, or have been designed as bench-top adjuncts only.^37,38,39,40,41^

The DRS system presented here shows suitability for use intraoperatively to aid margin assessment in cancer surgery. We developed a system with high spectral resolution and high data acquisition speed. As a result, we were able to scan a large area on the tissue and differentiate tissue in real time while creating minimal disruption to the surgical workflow. Differentiation was achieved as a result of distinct differences in the data between the mean spectra for normal and cancer tissue for the stomach and esophagus. The cause for the prominent difference in spectra, especially between a wavelength of 600 and 700 nanometers, for stomach and esophageal tissue is unclear but likely based either on tissue vascularity signals or on the specific quantities of different chromophores in each tissue.^42^ Further work is needed in this area.

A major limitation of ex vivo sampling is potential tissue degradation following resection and blood supply being cut off^43^; however, given that the entirety of the data acquisition protocol lasted approximately 10 minutes, we believe that the effect of this was minimized and tissue characteristics were preserved. Furthermore, the intuitive and easy-to-use graphical user interface we implemented would allow the surgeon to perform the sampling procedure within a timely manner in the operating room, preventing a substantial delay to the surgical procedure.

Compared with the current criterion standard of frozen sections, which can cost more than $3000 per patient to perform,^44^ our system has much lower costs, has a portable setup system (Figure 1), and does not require additional workforce. The system we have developed has a one-off cost of £8000 (US $9727), and given that it can be used for multiple sampling events, the per-patient costs would be much less. Our DRS system is also completely noninvasive and does not disrupt the tissue itself or interfere with its structure, allowing for a full analysis when sent to the histopathology department, especially important for small tumors. Nevertheless, the aim is for the DRS system to be integrated into the surgical workflow in the future as a device to assess tumor margins, with the intention to help reduce the burden on histopathology departments once the system has been fully developed, with less requirement for frozen section analysis.

Our method for real-time probe tracking has demonstrated high accuracy compared with ground truth and overcomes the point-based limitation that other DRS systems have faced.^26,29^ This enables the surgeon to follow the exact areas being sampled based on augmented data acquisition video feed. This tracking system together with the classification of each sample site on the tissue can be used to assess the resection margins of cancer intraoperatively. In this way, less healthy tissue could be removed, leading to a lower risk of morbidity for the patient. As such, the system has the potential to influence the intraoperative procedure and decision-making process.

### Limitations

This study has limitations. First, this is a single-center study, which limits the generalizability of its conclusions. A multicenter trial would help recruit participants from a wider population. Second, our method for correlation of tumor location on the specimen was limited by the histopathology protocols. Standard histopathological analysis of the specimens focused on the depth of the tumor to assess tissue invasion rather than the surface area of the cancer on the specimen, which was the basis of our sampling process. As a result, we had to use the hematoxylin-eosin slides together with the images of the sliced tissue and the images of the whole specimen to manually label tumor areas. This has potentially led to mislabeling of some of the data points on the border between the tumor and healthy tissue. Moreover, in this study, we excluded samples which were labeled as tumor but confirmed to be regressed tumor or fibrosis tissue. Given that this type of tissue is difficult to distinguish by a surgeon through tactile and visual assessment alone, it would be of great benefit to classify these types of tissue in future studies. Unfortunately, because of the sparsity of these samples, accurate analysis could not be made to include this data. Additionally, in our study, sampling was performed by 3 operators, which introduced interoperator variability with regard to sampling technique, potentially affecting the resulting DRS data. Probe angle should therefore be further explored to elucidate its effect on the results.

Lack of external validation is a major barrier to the safe implementation and routine use of artificial intelligence classification models in clinical practice.^45^ As such, external validation of our classification model on a separate cohort of patients is a crucial step toward the clinical translation of this technology. It is also vital that decision analysis studies and key stakeholder interviews are conducted to identify the optimal use-cases for the technology as well as the barriers and facilitators to its adoption in the future.^46,47^ The integration of the DRS technology into the surgical workflow to allow tumor mapping can be facilitated via augmented reality or an optional heatmap overlay added onto a laparoscopic or robotic camera feed, which constitutes only a minimal deviation from the standard plan of care.^48^

We have planned a concurrent in vivo validation of the real-time tracking technology and classification, for which adaptation of the probe tracking system will be needed. In the future, advanced deep learning neural networks can be used to help with accurate probe tip location detection and tracking. Moreover, correlation of the real-time tissue classification with clinical outcomes, such as recurrence, positive resection margin rate, or overall survival, will be required to show the clinical utility of this technology before its potential clinical adoption.^49^

## Conclusions

This study provides ex vivo validation of the DRS technology for real-time differentiation of gastric and esophageal cancer from healthy tissue using machine learning. As such, it is a step toward the development of a real-time in vivo tumor mapping tool. In the future, DRS technology and probe tracking need to be externally validated and tested in an intraoperative setting to assess their real-life utility for resection margin assessment and the potential of this technology in improving long-term outcomes.

## Notes

Supplement. **eFigure 1.** Photograph of Data Acquisition on an Oesophageal Specimen **eFigure 2.** Histopathological Analysis of the Specimen for the Purposes of Correlation of Suspected Tumour Locations
