# Supplementary material for: Real-time Tracking and Classification of Tumor and Nontumor Tissue in Upper Gastrointestinal Cancers Using Diffuse Reflectance Spectroscopy for Resection Margin Assessment
Source: JAMA Surg. 2022 Sep 7;157(11):e223899. doi: 10.1001/jamasurg.2022.3899 (PMC9453631; doi:10.1001/jamasurg.2022.3899)
Supplement: Supplement. — eFigure 1. Photograph of Data Acquisition on an Oesophageal Specimen eFigure 2. Histopathological Analysis of the Specimen for the Purposes of Correlation of Suspected Tumour Locations [file jamasurg-e223899-s001.pdf]

Supplemental Online Content

Nazarian S, Gkouzionis I, Kawka M, et al. Real-time tracking and classification of tumour and nontumor tissue in upper gastrointestinal cancers using diffuse reflectance spectroscopy for resection margin assessment. *JAMA Surg*. Published online September 7, 2022. doi:10.1001/jamasurg.2022.3899

eFigure 1. Photograph of Data Acquisition on an Oesophageal Specimen
eFigure 2. Histopathological Analysis of the Specimen for the Purposes of Correlation of Suspected Tumour Locations

This supplementary material has been provided by the authors to give readers additional information about their work.

© 2022 Nazarian S et al. *JAMA Surgery*.

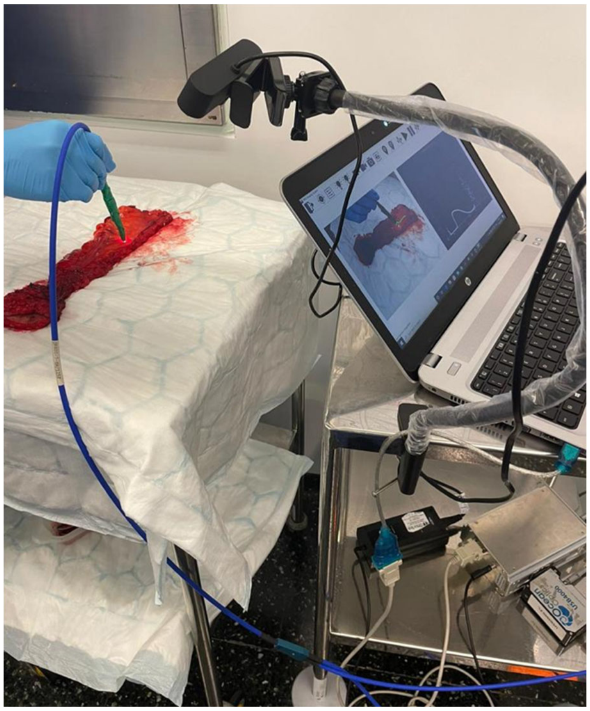

eFigure 1. Photograph of Data Acquisition on an Oesophageal Specimen

The DRS probe is used to sample the specimen whilst the spectral data is acquired on the graphical user interface.

© 2022 Nazarian S et al. *JAMA Surgery*.

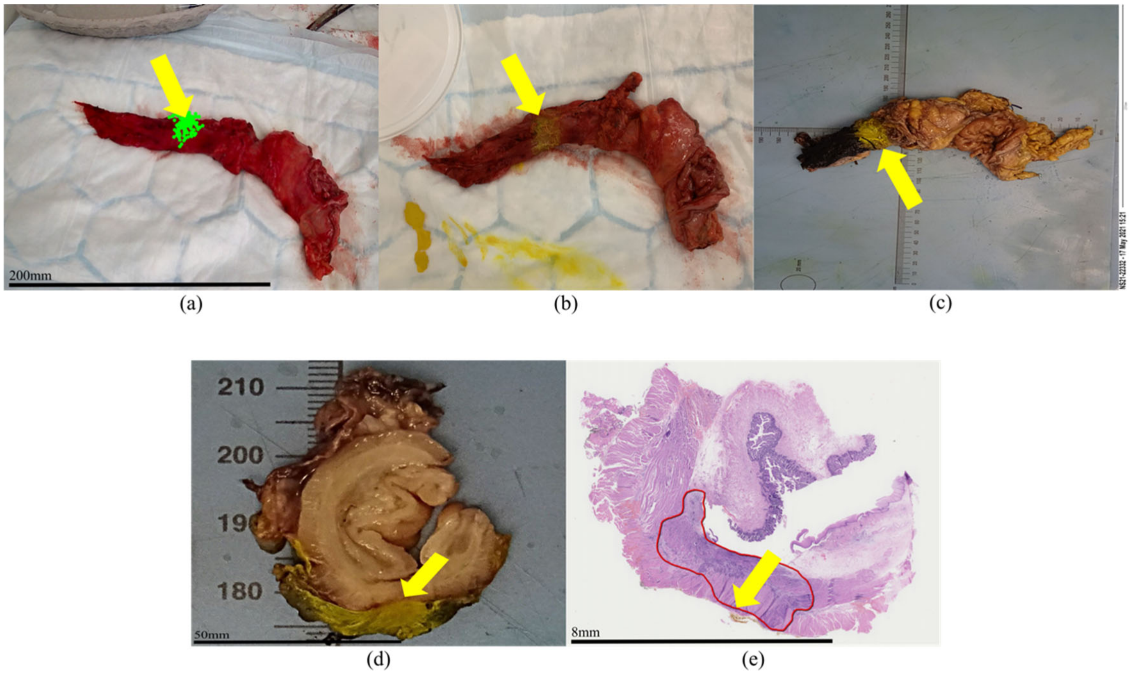

eFigure 2. Histopathological Analysis of the Specimen for the Purposes of Correlation of Suspected Tumour Locations

Histopathological correlation workflow is shown on an example of oesophageal adenocarcinoma. The correlation was performed using yellow tissue paint (marked with a yellow arrow across panels (a) – (e). (a) The ex vivo *specimen with tracked optical biopsy sites of the suspected 'tumour' area. (b)* Ex vivo *specimen following painting of the suspected 'tumour' tissue with yellow paint. (c) Specimen, having been placed in formalin, with yellow painted area visible. (d) Macroscopic slice of oesophageal tissue with the yellow painted area. (e) Microscopic H&E-stained slice with yellow paint (yellow arrow) and confirmed oesophageal adenocarcinoma (red outline).*

© 2022 Nazarian S et al. *JAMA Surgery*.
